# The Habit of Reticence

**Published:** 1904-07-30

**Authors:** 


### The Habit of Reticence.

Among the several responsibilities and duties attached to medical practice it is well recognised that there exists a special claim for the cultivation of the virtue of silence. The unreserved and whole-hearted self-revelation which it is the patient's interest to present can, indeed, only be obtained under the conviction and assurance that his confidences will be respected. Such conviction, therefore, forms the very basis of the relationship between the patient and his medical adviser, and anything which destroys or weakens it impairs in a proportionate degree the success and value of professional direction. Now it may be confidently claimed that a determined and wilful breach of the confidences which daily pass between patients and practitioners is an almost unknown experience. The standard of attainment in this respect is a very high one, and failure to conform to it of deliberate purpose can but rarely be charged against the profession. Even the most inexperienced practitioner feels, in addition to the promptings of self-respect, the force of habit and opinion adopted and practised by his fellows. But there is ever the danger that what one would never do of malice aforethought may occasionally occur, and with not less serious results, through carelessness or inattention, or, perchance, through too facile a response to the inquiries of good-natured busybodies. It is this risk which makes it advisable at times to urge the necessity for the unflagging cultivation of the habit of professional reticence. The man whose invariable practice it is never to talk about his patients' affairs, whether they be great or small, is not the man who in an unguarded moment lapses into some indiscreet remark, and thus betrays a trust committed to his care. Such a misfortune is reserved almost invariably for those whose habit is of the contrary order. They intend no harm, and yet they commit it. Insensibly they have prepared themselves for mischief, and what has seemed like harmless chatter about trifles has bred an overmastering habit that has paved the way for a breach of faith beyond the power of healing. That this is the true history of occasional departures from the honourable tradition of the profession will hardly be questioned. They are the consequence not of a wilful and wanton disregard of admitted obligations, but of a non-recognition of the principle that it is "the little foxes that spoil the vines." It is these considerations which render so urgent the claim that every practitioner shall cultivate, until it becomes part of his nature, a severe reticence regarding the pathological, the personal, and the domestic relationships of all persons who come under his professional care. And the greater the opportunities for speech the more rigid must be this discipline, for such enlarged opportunity means a greater area of possible prejudice both to the interests of the patient and the tone of the profession. That there is need to say these things may be found in two recent experiences. In one, a lecturer, speaking to a considerable audience of his professional colleagues, related details of the case of a well-known artist, mentioning a name probably familiar to every person in the room. The other may be found in an article in a leading medical journal in which a physician of unexceptional position permits himself to advance a speculation regarding the nature of the illness of a certain Royal personage. Even if no practical harm can be traced to such references and discussions, they are not the less to be condemned. No one will imagine for a moment that in the instances just quoted there was anything more than a desire to produce an apt and telling clinical illustration. But the ambition of a lecturer in this direction ought not to be attained at the expense of a custom which is ordered in the interest both of the public and the profession. The most distinguished, as well as the most obscure patient, is entitled to the protection of this custom, and his clinical and pathological individuality ought not to be dragged into the publicity of the lecture-room, however appropriate to the theme under discussion. Most of all, such references are to be deprecated because they argue a want of appreciation of professional obligation and delicacy, and set an example having an influence wholly pernicious. They weaken that practice of habitual reticence in which alone wisdom, honour, and safety are to be found.

